# Supersize panniculectomy: a case report

**DOI:** 10.1097/RC9.0000000000000499

**Published:** 2026-05-06

**Authors:** Stefano Uderzo, Roberta Boffo, Bruno Lo Sapio, Maria Maddalena Nicoletti, Alfredo Borriello, Mario Faenza

**Affiliations:** a Department of Plastic, Reconstructive and Aesthetic Surgery, Università degli Studi della Campania Luigi Vanvitelli, Ospedale del Mare, Napoli, Italy; b Department of Plastic, Reconstructive and Aesthetic Surgery, Ospedale del Mare, Napoli, Italy; c Department of Plastic, Reconstructive and Aesthetic Surgery, Università degli Studi della Campania Luigi Vanvitelli, Napoli, Italy; d Department of Plastic, Reconstructive and Aesthetic Surgery, Università degli Studi della Campania Luigi Vanvitelli, Napoli, Italy

**Keywords:** abdominoplasty, bariatric surgery, case report, panniculectomy, panniculus morbidus, plastic surgery

## Abstract

**Introduction and importance:**

Morbid obesity can present with life-limiting panniculus morbidus, a severe excess of abdominal tissue. We describe the importance of a multidisciplinary, tailored surgical approach involving the resection of 30 kg of tissue to achieve both functional and psychological recovery, despite high perioperative risks.

**Case presentation:**

A 47-year-old man [body mass index (BMI) 61] presented with a pendulous pannus extending to the knees, leading to urinary and sexual dysfunction, recurrent skin infections, ulcerations, and inability to perform daily activities. He underwent an elective, two-stage panniculectomy. Intraoperatively, two supraumbilical hernias and aberrant lymphovascular clusters were identified. A total of 30 kg of tissue was removed. Closure employed progressive-tension sutures and negative-pressure dressings. Postoperatively, he developed a seroma and transient urinary incontinence, both managed conservatively. By 1 month, he had regained independence in activities of daily living and demonstrated marked psychological improvement.

**Clinical discussion:**

Resection of 30 kg of pannus qualifies as a “supersize” panniculectomy. Common complications – seromas and wound dehiscence – are more frequent in patients with a high BMI and comorbidities such as diabetes and smoking. However, with meticulous planning and technique, this case shows that even high-risk patients can achieve meaningful functional restoration and quality-of-life gains.

**Conclusion:**

Amid the global obesity epidemic, this report advocates for a flexible, function-driven surgical strategy in extreme cases typically deemed ineligible for intervention. When needed, panniculectomy can deliver substantial benefits that outweigh the inherent risks.

## Introduction

Abdominoplasty is a key procedure for patients after significant weight loss, often post-bariatric. It improves appearance and alleviates functional and psychological issues related to excess skin. Identifying risk factors [high body mass index (BMI), skin excess, diabetes, smoking] is essential to minimize complications. Techniques include the classic bi-spinoiliac approach, “fleur-de-lis” with a vertical scar, reverse abdominoplasty, circumferential body lift, and lipoabdominoplasty. Main complications include seroma, scarring, and infection; systemic risks include pulmonary embolism and deep vein thrombosis^[^1–3^]^.

HIGHLIGHTS

- Panniculus morbidus may represent a functional indication for surgery prior to bariatric treatment.
- Supersize panniculectomy can restore mobility and hygiene in selected super-obese patients.
- Multidisciplinary perioperative management is essential to improve safety in high-risk cases.
- Standard and adjunctive surgical techniques reduce morbidity in massive pannus resection.
- Functional and quality-of-life benefits may outweigh surgical risks in advanced disease stages.

The Italian National Health System (SSN) usually covers abdominoplasty for post-bariatric patients with a BMI < 30 and stable weight for at least 6 months^[^1,3^]^. In selected cases, surgery may be approved due to complications from excess fat that alter surgical priority. In rare severe forms, obesity may evolve into “abdominal elephantiasis” or panniculus morbidus (PM). This report presents the diagnostic and therapeutic approach for one such case to support future clinical management. This work has been reported in line with the 2025 SCARE checklist^[^4^]^.

## Case presentation

### Patient information

The patient is a 47-year-old Caucasian man. His medical history includes diabetes mellitus and hypertension, which are treated with metoprolol. In 2010, he weighed 80 kg (height 1.75 m; BMI 26.1), but following psychosocial stress and prolonged night-shift work, his weight increased to 160 kg in 2016 (BMI 52). Several dietary and exercise attempts resulted in transient losses of 20–30 kg without lasting benefit and occurred in the absence of psychological support. He denied smoking, alcohol use, or illicit drug use.

### Clinical findings and timeline

Since 2021, the patient has reported progressive abdominal enlargement with the pannus extending to the knees, resulting in urinary and sexual dysfunction, impaired mobility, and an inability to work. Recurrent cutaneous infections and ulcerations have affected the most dependent portion, likely related to chronic lymphatic stasis and altered vascular drainage.

**Figure 1.**
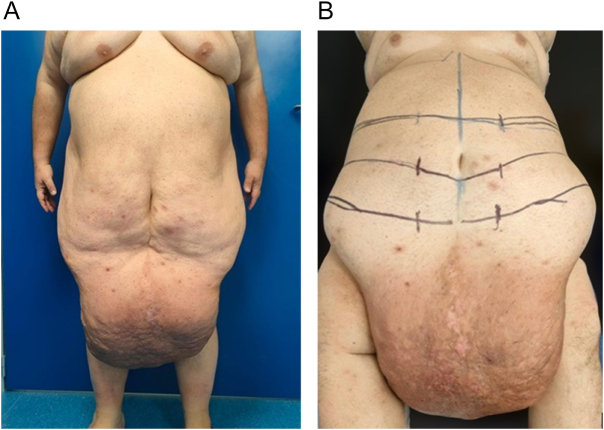
(A) Pre-op: preoperative front picture. (B) Markings: preoperative marks.

In December 2023, he presented to our clinic weighing 188 kg (BMI 61, super-obese class) (Fig. 1A). Physical examination revealed a markedly globose abdomen with massive adipose hypertrophy and a giant pannus. The dependent region was edematous, erythematous, indurated, malodorous, and showed multiple superficial ulcerations with serous exudation, without active bleeding. The skin displayed a characteristic “peau d’orange” appearance. Bowel sounds were preserved, Blumberg’s sign was negative, and deep palpation identified a small, non-tender peri-umbilical mass. Posture and gait were significantly compromised by the weight of the abdominal apron.

### Diagnostic assessment

Preoperative assessment included an abdominal CT scan, which revealed a massive subumbilical pendulous abdomen measuring 58 × 36 cm, associated with severe cellulitis and focal skin discontinuities due to ulceration. Based on these findings, the patient underwent surgical treatment in October 2024.

### Intervention

The surgical team was composed of the Chief of the Department and a senior attending surgeon, both Italian board-certified plastic surgeons, as well as two plastic surgery residents in their third and fourth years of training, respectively.

Preoperative drawing is shown in Figure 1B. The inferior marking consisted of a bi-spinoiliac line positioned approximately 7–8 cm above the root of the penis, corresponding to the planned final scar. Preoperative assessment was performed with the patient in the standing position, allowing gravity-dependent evaluation of the pannus to estimate the extent of resection. Superiorly, three lines were drawn: two representing alternative resection limits to be selected intraoperatively based on flap tension and tissue viability after pannus mobilization, and one inferior line delineating the dependent panniculus, laterally continuous with the inferior marking. The final extent of excision was adapted intraoperatively to balance maximal tissue removal with preservation of vascularity and minimization of wound complications.

From admission to the operating room, the patient wore anti-thrombosis compression stockings. The entire procedure was performed in a single operative session of 3 hours, and the main intraoperative steps are described below in chronological order.

The patient underwent general anesthesia. Urethral catheterization was difficult, as the penis had been completely concealed by the perineal fat. After surgical field preparation, anatomical landmarks were identified according to the preoperative markings.

The resection of the mass began on the right side and proceeded to the left through a skin incision using a No. 15 scalpel, followed by full-thickness electrocautery dissection from the lateral margins toward the midline, with contralateral displacement of the abdomen by the first operator.

Intraoperatively, multiple vascular-lymphatic structures greater than 0.5 cm in diameter, of uncertain origin and function, were identified. A urology consultation ruled out any involvement of the urogenital system, and these structures were presumed to correspond to circumflex arteries and veins.

**Figure 2.**
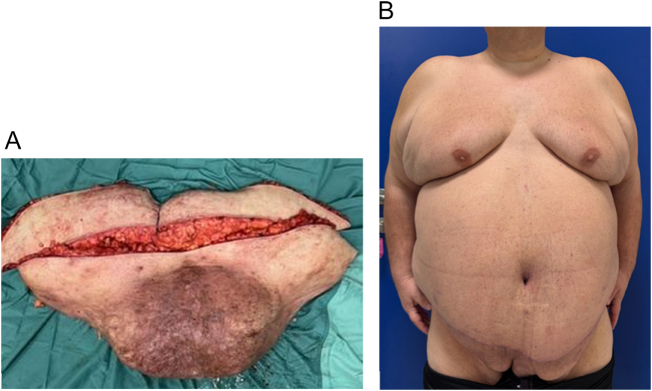
(A) Asportation: Mass excision. (B) Post-op: One-month postoperative front picture.

An initial adipose mass weighing 19.5 kg was excised, followed by the removal of an additional 9.5 kg of cutaneous-adipose tissue (Fig. 2A).

Umbilical isolation was performed by circumscribing the umbilicus and preserving its vascular pedicle. Minimal suprafascial dissection was carried out along the rectus muscles, intentionally limited to avoid extensive undermining, thereby reducing dead space and preserving flap vascularity.

Two supraumbilical hernias containing adipose tissue were reduced, and fascial reconstruction was carried out using 2-0 polypropylene sutures.

The upper abdominal flap was advanced caudally and secured using Baroudi sutures placed along the parasternal and hemiclavicular lines with 2-0 polyglactin sutures. Progressive tension sutures were placed in a linear and symmetrical fashion to reduce dead space and minimize seroma formation, ensuring adequate perfusion and appropriate anatomical positioning.

The umbilicus was repositioned with 3-0 polydioxanone subcutaneous sutures and interrupted 4-0 polypropylene skin sutures.

Two suprapubic closed-suction drains were placed. Layered closure was achieved using deep 2-0 Monosyn sutures and superficial subcutaneous and intradermal closure with 3-0 Monosyn. Hemostatic glue was applied, and dressing was completed with PICO negative-pressure wound therapy combined with an elastic-compressive bandage for external compression to reduce edema, support wound healing, and decrease seroma risk.

Following an overnight stay in the intensive care unit, during which the patient underwent extubation, non-invasive ventilation, and analgo-sedation, he was admitted to the plastic surgery ward for 5 days. Postoperative laboratory tests revealed a hemoglobin decrease to 9.2 g/dl without a transfusion requirement, transient neutrophilic leukocytosis resolving within 24 hours, and hyperglycemia. Ceftriaxone, linezolid (600 mg), intravenous acetaminophen, fluid therapy, and enoxaparin sodium 8000 IU once daily were administered. Drains were removed on postoperative day 10 during outpatient follow-up, with total output below 50 ml.

### Follow-up and outcomes

The patient's home therapy consisted of Enoxaparin sodium 6000 IU, 1 fl subcutaneous for 10 days; Ceftriaxone 1 g, twice per day for 5 days; and Bromelain (anti-edema supplement) taken by mouth for 20 days.

Negative-pressure wound therapy and compressive dressing were removed after seven days and replaced with standard adhesive dressings and an abdominal compression garment for 1 month.

During outpatient follow-ups, the patient reported that urinary incontinence had resolved following urologic consultation and developed a peri-umbilical seroma of less than 100 cc, which was drained in two outpatient sessions and attributed to predictable liponecrosis (Fig. 2B).

Additional intraoperative and postoperative images are available as supplementary material (Supplemental Digital Content Figure 1, available at: http://links.lww.com/IJSCR/A46; Supplemental Digital Content Figure 2, available at: http://links.lww.com/IJSCR/A47).

## Discussion

In body contouring surgery, abdominoplasty does not correspond to a single standardized procedure but rather to a spectrum of techniques selected according to skin laxity, fat excess, and muscular involvement. As described by Matarasso, treatment may range from isolated liposuction or mini-abdominoplasty in mild cases to full abdominoplasty with umbilical transposition and muscular plication in more advanced conditions. In post-bariatric or complex patients, extended techniques such as fleur-de-lis abdominoplasty, circumferential body lift, or panniculectomy may be required^[^2^]^.

Abdominoplasty is usually performed after significant weight loss, typically following bariatric surgery, which represents the first-line treatment for severe obesity. In this case, however, advanced local disease with infection, ulceration, functional impairment, and anesthesiological risk related to the mass precluded bariatric surgery as an initial option, making our operation a necessary functional bridging procedure to restore hygiene, mobility, and allow future metabolic treatment rather than an esthetic intervention.

In the literature, the condition known as PM is described as a massive accumulation of hanging adipocutaneous tissue in the lower abdomen, typically associated with chronic lymphedema and venous obstruction due to the weight of the pannus itself, leading to persistent inflammation, collagen deposition, tissue fibrosis, and development of the characteristic “peau d’orange” appearance^[^5,6^]^.

Our patient corresponded to Grade V according to the American Society of Plastic Surgeons' pannus classification system, which denotes a caudal extension of the panniculus reaching the knees or extending beyond them^[^7^]^.

A review of the literature shows substantial consistency in surgical strategies for severe PM. Most authors plan flexible superior resection margins to prevent flap tension, use suspension devices such as Steinmann pins when manual mobilization is impractical, and limit superior flap dissection by avoiding elevation to the xiphoid process to reduce dead space and seroma formation^[^8–10^]^.

This procedure can be classified more appropriately as a panniculectomy rather than an abdominoplasty. The term “supersize panniculectomy” has been introduced to describe resections exceeding 10 kg, with caudal extension to the mid-thigh or below. In a retrospective series involving 26 patients, the mean excised pannus weight was 15.6 kg, with a relatively low complication rate, most events being treated conservatively. It is worth noting that, in the majority of reported cases, panniculectomies are substantially less extensive, with most authors describing significantly smaller resections and total pannus weights commonly well below 30 kg^[^10^]^. In our patient, approximately 30 kg of adipocutaneous tissue, both pathological and redundant, were removed, placing this case among the upper range of published experiences.

At wound closure, some teams adopted multilayer suturing techniques, while others favored vacuum-assisted closure therapy with healing by secondary intention^[^11^]^. Our surgical strategy aligns closely with these established technical principles.

Only a limited number of comparable extreme cases are available in the literature. Paul *et al* reported a 54-kg panniculectomy in a 317-kg diabetic patient, with a surgical duration of 7 hours and transfusion of six units of blood^[^12^]^. More recently, William *et al* described the excision of 94 kg of panniculus in a 244-kg Hispanic male in Texas, illustrating both the technical challenges and physiological limits of surgical management in end-stage obesity^[^13^]^. These cases emphasize that, although rare, such interventions may be necessary in selected patients when conservative or conventional strategies prove inadequate.

As with any major operation, panniculectomy and abdominoplasty carry inherent risks. Common local complications include seroma, wound dehiscence, infection, hematoma, liponecrosis, and skin necrosis, while serious systemic events mainly involve deep vein thrombosis and pulmonary embolism. Long-term effects may include sensory impairment and pathological scarring. Smoking and diabetes are well-established surgical risk factors, as they impair microcirculation, immune response, and wound healing^[^3^]^.

In addition, postoperative risk has been shown to correlate not only with body mass index and the volume of resected tissue but also with specific demographic and procedural variables. Notably, each 10-year increase in age raises the complication risk by a factor of 1.270; male patients present a 2.111-fold higher risk compared to females; and for every additional 500 g of resected tissue, the likelihood of complications increases by a factor of 1.099^[^14^]^.

Consistently, a large retrospective study on post-bariatric abdominoplasty reported an overall complication rate of 29.8%, with wound dehiscence (14.6%) and seroma formation (8.7%) being the most frequent adverse events. Identified risk factors included elevated preoperative BMI, diabetes, smoking, and prolonged intervals between bariatric surgery and reconstructive procedures, while optimization of body weight and comorbidities demonstrated a protective effect against postoperative morbidity^[^1^]^.

Despite the inherent surgical risks, the literature consistently shows that improvements in quality of life largely outweigh postoperative morbidity in patients affected by PM^[^6,8–11^]^.

Our experience supports this conclusion, corroborating that functional restoration, regained autonomy, and psychological benefit justify surgical intervention, even in high-risk settings.

Although once regarded as an exceptional and end-stage manifestation of obesity, accumulating evidence indicates that PM is becoming increasingly prevalent worldwide in parallel with the global rise in severe obesity. Consequently, cases once considered extraordinary are expected to become more frequent in daily surgical practice, underscoring the need for early diagnosis, appropriate referral, and standardized multidisciplinary management pathways^[^12^]^.

Taken together, this report illustrates the management of an advanced case of PM through a multidisciplinary surgical strategy, highlighting both functional and psychological recovery while acknowledging intrinsic limitations related to single-patient design and short follow-up, counterbalanced by the clinical relevance of the surgical approach and its multidisciplinary implications.

This case reinforces that, in selected patients, panniculectomy may represent a necessary bridging intervention rather than a deviation from standard obesity care, restoring functional capacity and enabling future metabolic treatment.

## Conclusion

This case supports panniculectomy as a functional bridging procedure in selected patients with extreme obesity when bariatric surgery cannot be safely offered as a first-line treatment. In this case, the combination of standard surgical techniques with less conventional measures, integrated within a multidisciplinary framework and supported by structured perioperative protocols, contributed to reducing the risk of prolonged hospitalization and significantly accelerated early postoperative recovery, allowing for a more timely and safer patient discharge. In a world where the so-called globesity is increasing day by day and these extreme cases are becoming more common in our ORs, this case report enriches the scientific landscape by providing a model for complex case management in surgery and may contribute to improving future clinical practice.

## Strengths and limitations

This case report highlights the management of a rare and extreme condition of PM through a multidisciplinary, staged surgical approach, showing the functional and psychological benefits of panniculectomy. Limitations are: single patient and short follow-up. Strengths are: surgical strategy, risk management, and cross-specialty relevance in treating advanced obesity-related complications.

## Data Availability

All relevant data are included within the article. Additional clinical details are available from the corresponding author upon reasonable request, in compliance with patient confidentiality.
